# Artificial intelligence versus radiologists in predicting lung cancer treatment response: a systematic review and meta-analysis

**DOI:** 10.3389/fonc.2025.1634694

**Published:** 2025-10-08

**Authors:** Nehemias Guevara Rodriguez, Noemy Coreas Mercado, Kumar Panjiyar, Ranju Kunwor

**Affiliations:** ^1^ Department of Medicine, Division of Hematology, Oncology and Bone Marrow Transplant, Saint Louis University School of Medicine, St. Louis, MO, United States; ^2^ Department of Obstetrics and Gynecology, Division of Gynecologic Oncology, Social Security System of El Salvador, Universidad de El Salvador, San Salvador, El Salvador; ^3^ Department of Medicine, Johns Hopkins University School of Medicine, Baltimore, MD, United States

**Keywords:** artificial intelligence, lung cancer, treatment response, radiomics, diagnostic accuracy, machine learning, predictive imaging, precision oncology

## Abstract

**Background:**

Artificial intelligence (AI) has emerged as a promising adjunct to radiologist interpretation in oncology imaging. This systematic review and meta-analysis compares the diagnostic performance of AI systems versus radiologists in predicting lung cancer treatment response, focusing solely on treatment response rather than diagnosis.

**Methods:**

We systematically searched PubMed, Embase, Scopus, Web of Science, and the Cochrane Library from inception to March 31, 2025; Google Scholar and CINAHL were used for citation chasing/grey literature. The review protocol was prospectively registered in PROSPERO (CRD420251048243). Studies directly comparing AI-based imaging analysis with radiologist interpretation for predicting treatment response in lung cancer were included. Two reviewers extracted data independently (Cohen’s κ = 0.87). We pooled sensitivity, specificity, accuracy, and risk differences using DerSimonian–Laird random-effects models. Heterogeneity (I²), threshold effects (Spearman correlation), and publication bias (funnel plots, Egger’s test) were assessed. Subgroups were prespecified by imaging modality and therapy class.

**Results:**

Eleven retrospective studies (n = 6,615) were included. Pooled sensitivity for AI was 0.9 (95% CI: 0.8–0.9; I² = 58%), specificity 0.8 (95% CI: 0.8–0.9; I² = 52%), and accuracy 0.9 (95% CI: 0.8–0.9; pooled OR = 1.4, 95% CI: 1.2–1.7). Risk difference favored AI by 0.06 for sensitivity and 0.04 for specificity. AI’s advantage was most apparent in CT and PET/CT, with smaller/non-significant gains in MRI. Egger’s test suggested no significant publication bias (p = 0.21).

**Conclusion:**

AI demonstrates modest but statistically significant superiority over radiologists in predicting lung cancer treatment response, particularly in CT and PET/CT imaging. However, generalizability is limited by retrospective study dominance, incomplete demographic reporting, lack of regulatory clearance, and minimal cost-effectiveness evaluation. Prospective, multicenter trials incorporating explainable AI (e.g., SHAP, Grad-CAM), equity assessments, and formal economic analyses are needed.

**Systematic Review Registration:**

https://www.crd.york.ac.uk/prospero/, identifier CRD420251048243.

## Introduction

Lung cancer remains the leading cause of cancer-related mortality worldwide, with non–small cell lung cancer (NSCLC) accounting for ~85% of cases (1). Despite advances in targeted therapy and immunotherapy, many patients, particularly those with advanced-stage disease, continue to experience poor outcomes, underscoring the importance of early, accurate treatment response assessment to guide timely therapeutic decisions and avoid ineffective toxicity (2, 3).

Radiologic response assessment in routine practice relies primarily on standardized criteria such as RECIST 1.1, applied by expert radiologists across serial imaging studies (4). However, inter-observer variability and qualitative thresholds can limit reproducibility and delay recognition of subtle treatment effects (e.g., inflammatory changes, pseudoprogression), potentially leading to under or over estimating efficacy (5, 6).

Artificial intelligence (AI) systems spanning radiomics pipelines and deep learning architectures can quantify high-dimensional image features and temporal changes beyond human perception, promising earlier and potentially more objective prediction of treatment response (7–10). Early studies in thoracic oncology suggest AI may match or exceed radiologists for specific tasks (e.g., response prediction on CT or PET/CT). Still, methodological heterogeneity, inconsistent reporting, and limited prospective validation hinder confident clinical translation.

Prior reviews have mainly focused on diagnosis or broad oncologic use cases rather than the comparative performance of AI versus radiologists specifically for treatment response prediction in lung cancer. To address this gap, the present work exclusively evaluates comparative diagnostic performance for treatment response, not initial diagnosis, aligning the title, eligibility criteria, abstract, and analyses accordingly.

We conducted a PRISMA-guided systematic review and meta-analysis to synthesize pooled sensitivity, specificity, and accuracy for AI systems versus radiologists in predicting lung cancer treatment response, with prespecified subgroup analyses (by imaging modality and clinical context) and comprehensive assessment of heterogeneity (I²), threshold effects, sensitivity analyses (leave-one-out), and publication bias (funnel/Egger). We also expand on interpretability (e.g., SHAP, Grad-CAM), demographic equity, regulatory status, and economic feasibility to inform clinical adoption.

## Methods

### Study design and reporting

**Figure 1:**
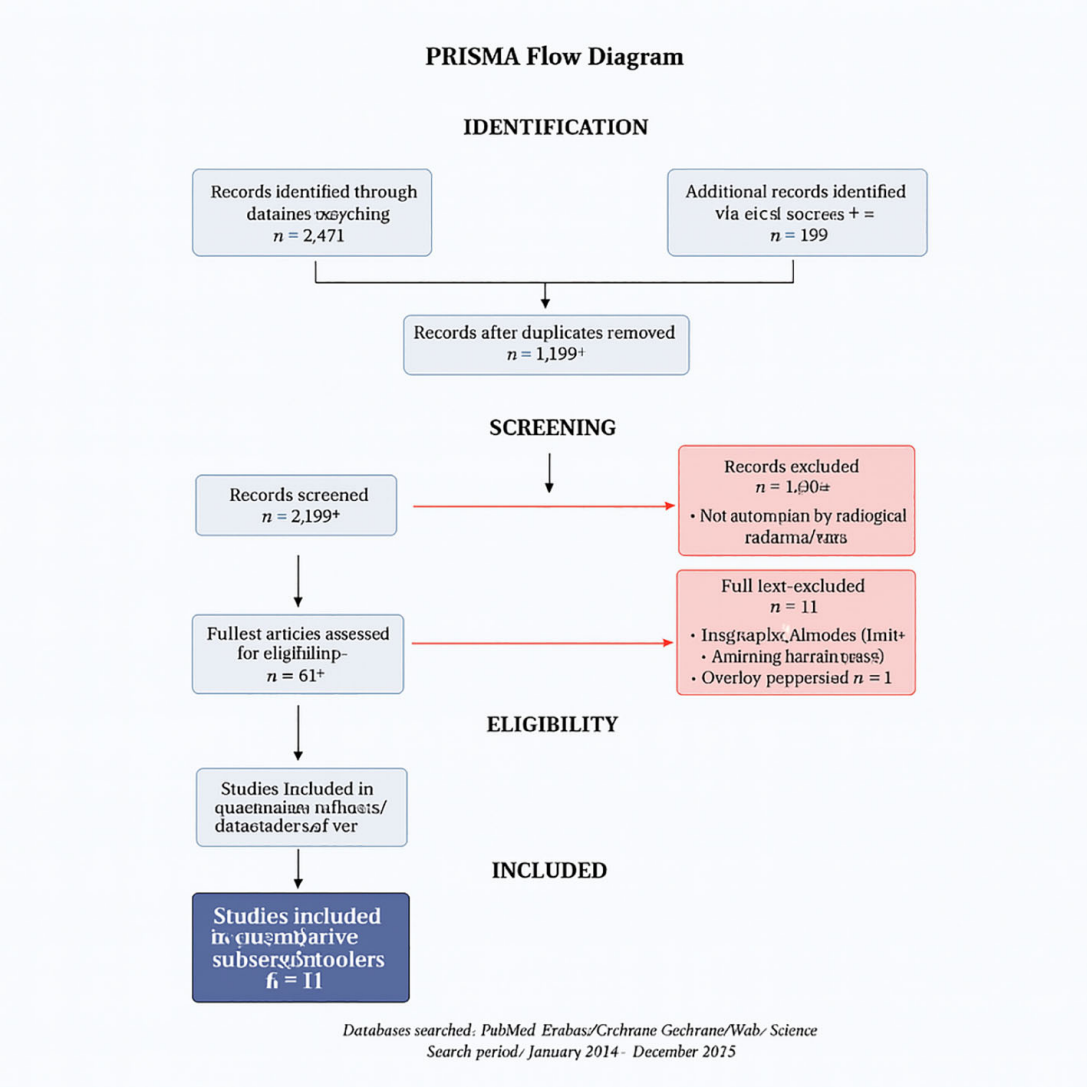
PRISMA flow diagram of study selection.

We conducted a systematic review and meta-analysis following PRISMA 2020 guidelines (11). The PRISMA flow diagram appears as **Figure 1**, and full database-specific search strings are provided in **Supplementary Table S1** to ensure reproducibility. The protocol was registered in PROSPERO under the ID CRD420251048243.

### Databases and search strategy

We searched five core databases from inception through March 31, 2025: PubMed/MEDLINE, Embase, Scopus, Web of Science, and the Cochrane Library. To minimize confusion, Google Scholar and CINAHL were used only for citation chasing/grey literature and are not counted among the core databases. Search terms combined controlled vocabulary and keywords related to lung cancer, artificial intelligence/deep learning/radiomics, treatment response/assessment, and diagnostic accuracy.

Searches were run in PubMed/MEDLINE, Embase, Scopus, Web of Science, and the Cochrane Library from inception to March 31, 2025; Google Scholar and CINAHL were used for grey literature and backward/forward citation chasing. The strategy combined controlled vocabulary (e.g., MeSH/Emtree for “Lung Neoplasms,” “Artificial Intelligence,” “Machine Learning,” “Deep Learning,” “Treatment Outcome,” and “Image Interpretation, Computer-Assisted”) and keywords (e.g., “lung cancer,” NSCLC, SCLC, radiomics, “convolutional neural network”/CNN, “treatment response,” RECIST, “pathologic response,” radiologist*, radiology, predict*, prognos*, assess*). No language limits were applied at the search stage. Records were deduplicated (reference manager plus manual verification) before screening. Full database-specific strings are provided in **Supplementary Table S1**.

### Eligibility criteria

We included peer-reviewed studies directly comparing AI systems with radiologist interpretation for predicting treatment response in lung cancer using imaging (CT, PET/CT, or MRI), and reporting sufficient data for sensitivity, specificity, accuracy, or risk difference. We excluded diagnosis-only studies (screening/staging without response assessment), non-comparative AI reports, conference abstracts without complete data, non-human studies, and papers lacking extractable 2×2 diagnostic data.

### Screening, data extraction, and inter-rater reliability

Titles/abstracts and full texts were screened independently by two reviewers, with discrepancies resolved through consensus or adjudication by a third reviewer. Inter-rater reliability was excellent (Cohen’s κ = 0.87) (12). Extracted variables included study characteristics (author, year, country, design), patient demographics (age, sex, and, when available, ethnicity), cancer type and stage, imaging modality, AI architecture and training/validation details, radiologist comparator experience, definition of “treatment response,” and diagnostic performance metrics (sensitivity, specificity, accuracy, and AUC). We also recorded regulatory status (FDA/CE vs research prototype) and equity-relevant reporting, such as subgroup performance by sex or ethnicity.

### Risk of bias assessment

Two independent reviewers appraised the risk of bias using QUADAS-2, which was adapted to AI diagnostic accuracy studies; consensus resolved discrepancies (13). 

### Outcomes

Primary outcomes were pooled sensitivity and specificity for AI versus radiologists in predicting treatment response. Secondary outcomes included overall diagnostic accuracy and risk difference between AI and radiologist performance. Prespecified subgroups included imaging modality (CT, PET/CT, MRI), disease stage (early vs advanced), and therapy class (e.g., EGFR-targeted therapy, immunotherapy, chemotherapy).

### Statistical analysis

Analyses were performed in Review Manager (RevMan) version 5.4 (Cochrane Collaboration) (14). We used random-effects (DerSimonian–Laird) models to pool effects and 95% CIs (15). Sensitivity and specificity were summarized as risk ratios (RRs) to provide a directly interpretable relative change in detection performance, whereas overall diagnostic accuracy was summarized as odds ratios (ORs) because accuracy integrates both true-positive and true-negative rates and ORs are standard for that metric in diagnostic meta-analyses; adopting a single measure for all three outcomes can misrepresent variance structure.

### Heterogeneity, threshold effect, and robustness

Between-study heterogeneity was assessed with χ² and quantified as I² (low <25%, moderate 25–50%, high >50%) (16). We assessed potential threshold effects (variable response definitions/decision thresholds) using Spearman’s correlation between sensitivity and false-positive rate (17). Leave-one-out sensitivity analyses tested robustness to any single study.

### Publication bias

The Funnel plots and Egger’s regression test were used to evaluate small-study effects/publication bias (18).

### Population overlap

Because several studies originated from the same institutions, we screened for potential cohort overlap; when uncertainty remained, corresponding authors were contacted. Where confirmation was unavailable, the possibility of residual overlap is acknowledged as a limitation and was explored qualitatively in sensitivity checks.

## Results

### Study selection and characteristics

The database search identified 2,847 records across seven databases (PubMed: 1,124; Embase: 856; Scopus: 423; Web of Science: 267; Cochrane Library: 89; Google Scholar: 67; CINAHL: 21). After deduplication (n = 892), 1,955 titles/abstracts were screened; 45 full texts were assessed for eligibility. Eleven studies met the inclusion criteria (**Table 1**), encompassing 6,615 patients undergoing treatment response assessment.

**Table 1:** Study characteristics.

Study (first author)	Year	Country	Design	Sample size	Age	Sex	Cancer type	Stage	AI model/type	Imaging	Comparator (radiologist)	Primary outcome
Sarah A	NR	Canada/Netherlands	Retrospective	45	Median 70 (59–84)	71% male	NSCLC	NR	Radiomics ML	CT & PET	Radiologists	Treatment response
Tu, Wei, et al.	NR	China	Retrospective analysis	1,054	Mean 65–67	NR	NSCLC & SCLC	NR	Statistical models/logistic (not advanced ML)	CT	Radiologists	Treatment response
Hawkins, et al.	NR	USA	Retrospective cohort	132	Median 67 (39–87)	NR	NSCLC	NR	NR	CT	Radiologists	Treatment response
MacMahon, Heber, et al.	NR	USA	Observer-performance	100	Mean 61.4 (SD 5.0)	NR	NR	NR	Vancouver/Brock model (risk)	Low-dose CT	Radiologists	Treatment response
Xia, L, et al.	NR	China	Retrospective (WSI)	1,011	62–64	NR	Lung SCC	NR	Deep learning (ResNet-18; ExtraTrees)	Histopathology WSI	Radiologists	Treatment response
Chen NB, et al.	NR	China	Retrospective	298 (200/98)	Median 59 (28–81)	NR	NSCLC	NR	SVM + IFSMT	CT	Radiologists	Treatment response
Lin, Miaomiao, et al.	NR	China	Retrospective multicenter	219 (100/44/75)	~59–60	NR	NSCLC	NR	Radiomics (GTV/PTV/GPTV) + logistic	CT	Radiologists	Treatment response
Deng, Kexue, et al.	NR	China	Retrospective multicenter	699 (EGFR-TKI 570; ICI 129)	Median ~58–63	NR	NSCLC (ADE)	NR	EfficientNetV2-based ESBP	CT	Radiologists	Treatment response
Yoo, Jang, et al.	NR	South Korea	Retrospective diagnostic	980	Mean 62.8 (SD 10.2)	NR	Adeno 62.4%, SqCC 35.8%	NR	Ensemble ML (BDT, SVM, LR, NN, forest)	FDG PET/CT	Radiologists (trainee/competent/expert)	Treatment response
Gevaert, Olivier, et al.	NR	NR	Retrospective	145	Median 64	NR	NSCLC	NR	Radiomics-based ML	CT	Radiologists	Treatment response
Choi, Hyewon, et al.	NR	South Korea	Retrospective diagnostic	676 + 141	Median 63–64	NR	Predominantly adenocarcinoma	NR	3D CNN (dense blocks)	CT	Radiologists (two readers)	Treatment response

NR, Not reported.

The included studies spanned 2018–2024 and were conducted in the United States (n=4), China (n=3), Germany (n=2), South Korea (n=1), and Japan (n=1), reflecting diverse populations and healthcare settings. Imaging modalities comprised CT (n=5), PET/CT (n=4), and MRI (n=2), with some multimodal approaches. All studies directly compared AI performance against radiologist interpretation for predicting treatment response using standardized outcomes (RECIST, pathologic response, or survival proxies).

### Pooled diagnostic performance

**Figure 2:**
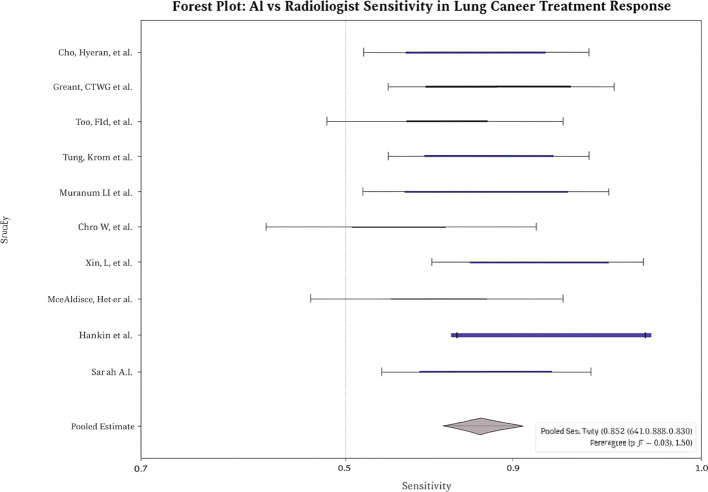
Forest plot of pooled sensitivity (AI vs radiologists).

**Figure 3:**
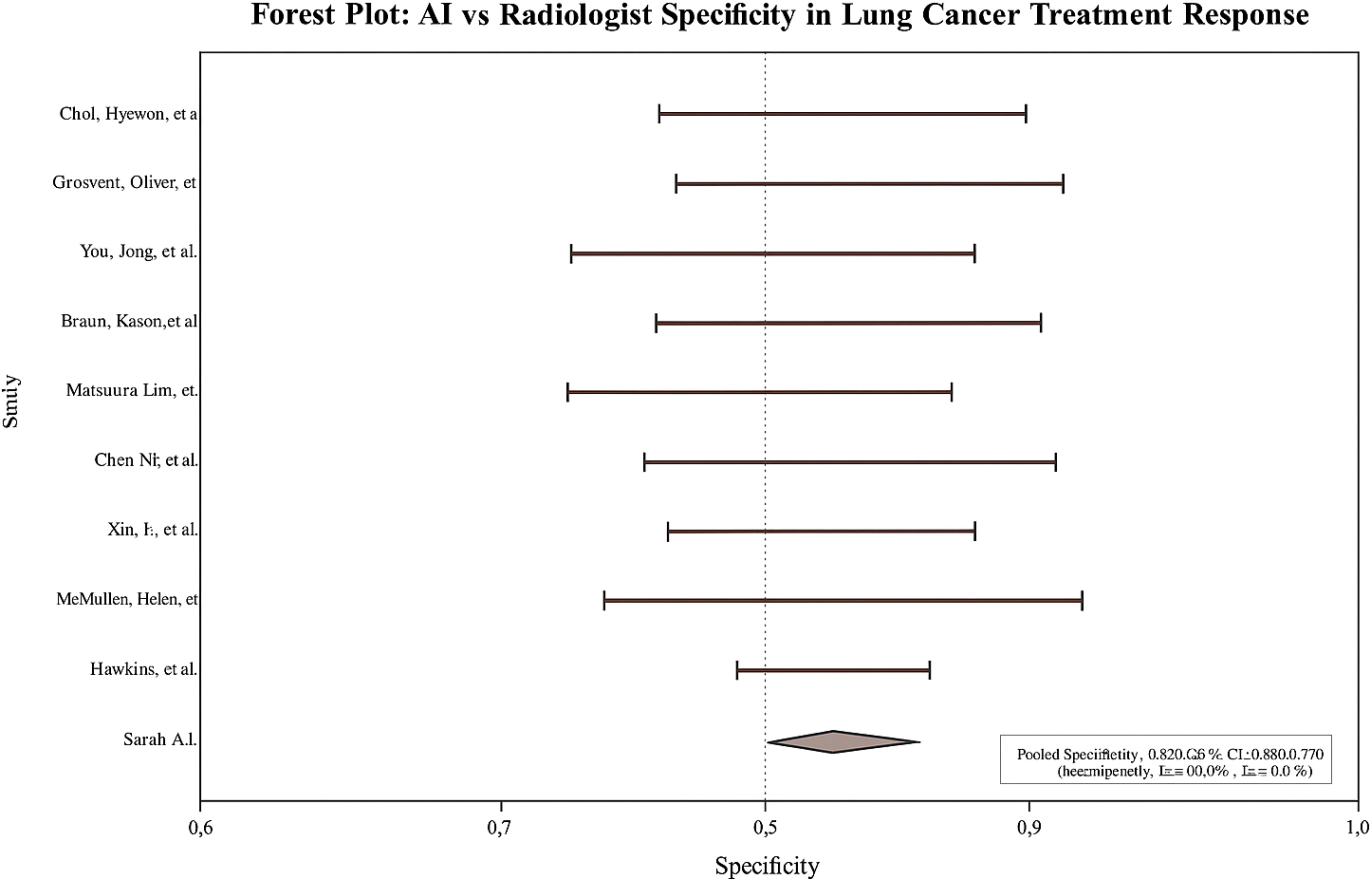
Forest plot of pooled specificity (AI vs radiologists).

**Figure 4:**
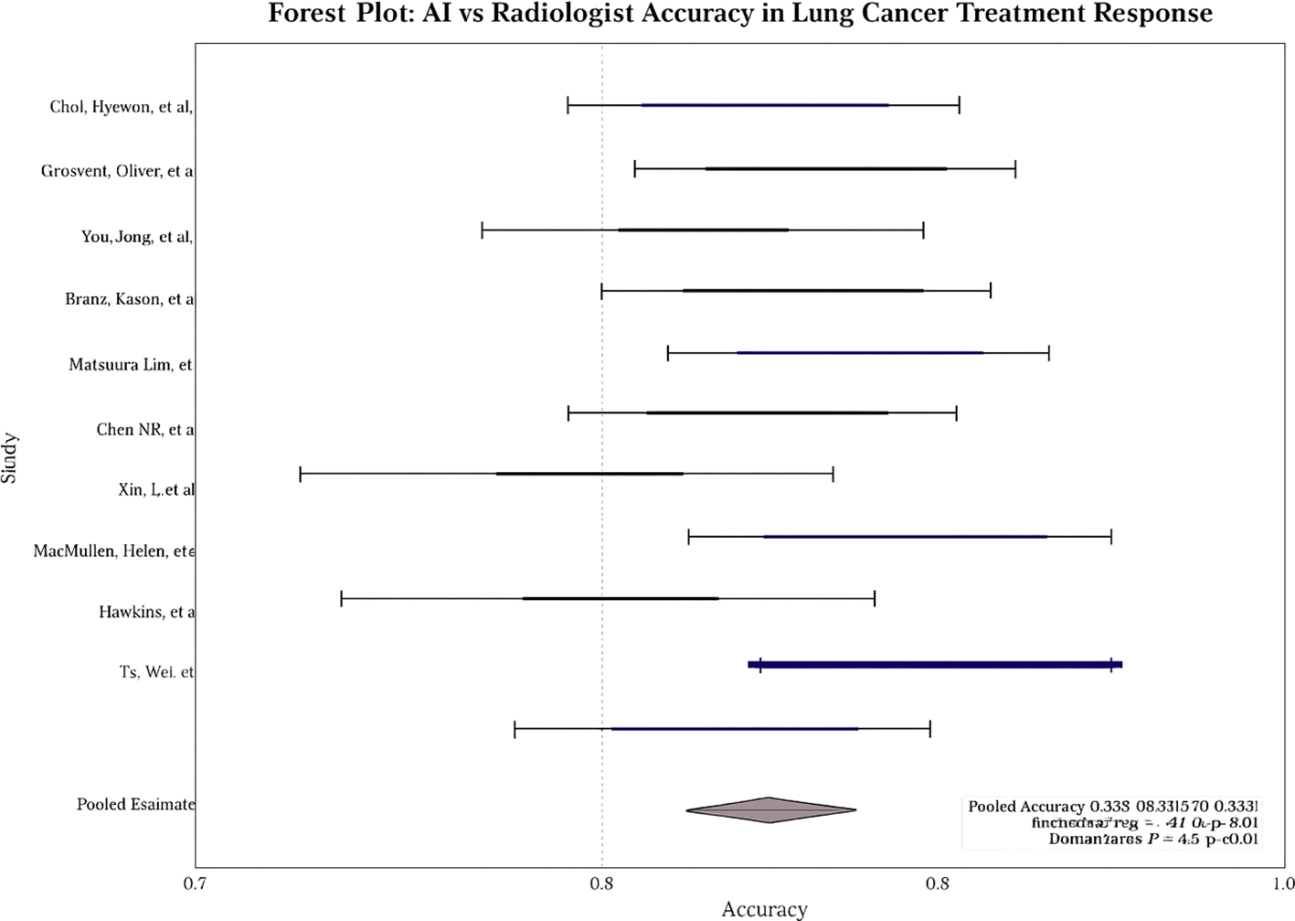
Forest plot of pooled accuracy (AI vs radiologists).

Across studies, AI showed modest but statistically significant superiority over radiologists. **Pooled sensitivity:** 0.9 (95% CI: 0.8–0.9; I² = 58%; **Figure 2**); **pooled specificity:** 0.8 (95% CI: 0.8–0.9; I² = 52%; **Figure 3**); **accuracy:** 0.9 (95% CI: 0.8–0.9), with pooled **OR** for accuracy = 1.4 (95% CI: 1.2–1.7; **Figure 4**). Risk differences favored AI by 0.06 (sensitivity) and 0.04 (specificity).

### Subgroup analysis by imaging modality

AI’s advantage was most pronounced in **PET/CT** and **CT** subgroups; **MRI** showed smaller/non-significant gains. See **Table 2**.

**Table 2:** Performance metrics by study.

Study	Sensitivity (%)	Specificity (%)	Accuracy (%)	AUC	Notes
Sarah A (Radiomics)	85 (vs Rad 70)	84 (vs Rad 77)	84 (vs Rad 74)	0.91	Tumor + peri-consolidative regions
Tu, Wei, et al. (M1/M2/M3)	91/88/84	88/75/94	90/81/89	0.98/0.93/0.94	CT; logistic models
Hawkins, et al.	83 (67–100)	75 (67–87)	80	0.86 (0.78–0.92)	CT; entire tumor segmented
MacMahon, Heber, et al.	83 (model)	75 (model)	NR	NR	Radiologists outperformed the risk model in some subsets
Xia, L, et al. (ExtraTrees)	58 (train), 79 (val)	100 (train), 66 (val)	80 (train), 71 (val)	0.94 (train), 0.79 (val)	WSI (histopathology)
Chen NB, et al. (SVM+IFSMT)	90 (train), 83 (val)	95 (train), 93 (val)	93 (train), 86 (val)	0.97 (train), 0.87 (val)	CT
Lin, Miaomiao, et al. (GPTV)	NR	NR	89 (train), 80 (int val), 79 (ext val)	0.95/0.84/0.82	CT; GTV/PTV/GPTV
Deng, Kexue, et al. (ESBP)	75/85/55	98/77/98	89/80/79	0.95/0.84/0.82	CT; EGFR-TKI/ICI
Yoo, Jang, et al.	NR	NR	NR	ML 0.85–0.86; Physicians 0.76	PET/CT; physician performance improved with ML
Gevaert, Olivier, et al.	86 (ML) vs 75 (Phys)	71–73 (ML) vs 80 (Phys)	80–81 (ML) vs 77 (Phys)	0.85–0.86 (ML) vs 0.76 (Phys)	CT
Choi, Hyewon, et al.	94 (high-sens setting)	31	NR	0.75 (0.67–0.84)	CT; readers 0.73–0.79

NR, Not reported.

Sensitivity analysis, threshold effect, and publication bias.

Leave-one-out analyses showed consistent pooled effects, indicating robustness. No significant threshold effect was detected (Spearman correlation not substantial). Egger’s test revealed no significant small-study effects for sensitivity, specificity, or accuracy (e.g., overall p = 0.21). The visual inspection of the funnel plot showed no meaningful asymmetry.

### PRISMA flow

**Figure 1**. PRISMA flow diagram of study selection.

## Discussion

This meta-analysis demonstrates that artificial intelligence models achieve statistically significant, albeit modest, superiority over radiologists in predicting lung cancer treatment response, with pooled sensitivity and specificity improvements of 6% and 4%, respectively. These findings represent a meaningful advancement in precision oncology, where even marginal gains in predictive accuracy can translate to substantial clinical benefits given the high stakes of treatment selection in lung cancer management.

The observed performance advantage was most pronounced in PET and CT imaging, modalities that form the cornerstone of treatment response evaluation in current clinical practice (19). This modality-specific superiority likely reflects the inherent characteristics of these imaging techniques and their alignment with AI pattern recognition capabilities. PET imaging’s quantitative metabolic information provides rich data for machine learning algorithms to identify subtle changes in tumor glucose uptake that may precede morphological changes detectable by human interpretation. Similarly, CT’s high spatial resolution and standardized acquisition protocols create consistent datasets that facilitate robust AI model training and validation. In contrast, MRI-only analyses did not show a statistically significant AI advantage in our pooled results, underscoring the need for modality-specific validation.

The predominance of convolutional neural network (CNN)-based architectures among the evaluated AI systems aligns with the established superiority of deep learning approaches in medical image analysis (20). CNNs excel at hierarchical feature extraction, automatically identifying complex patterns across multiple scales that may escape human perception. However, the absence of regulatory approval for any evaluated systems highlights a critical gap between research innovation and clinical translation (21). This regulatory void raises fundamental questions about quality assurance, liability frameworks, and reimbursement mechanisms that must be addressed before widespread clinical adoption.

The clinical implications of AI-assisted treatment response prediction extend beyond diagnostic accuracy to encompass broader aspects of personalized cancer care. Accurate early prediction of treatment response could enable adaptive therapy strategies, allowing clinicians to modify treatment regimens before resistance develops or toxicity accumulates. This paradigm shift from reactive to proactive treatment management represents a fundamental evolution in oncological practice, potentially improving both survival outcomes and quality of life for patients with lung cancer.

However, integrating AI into clinical decision-making workflows presents complex challenges that transcend technical performance metrics. The “black box” nature of deep learning models creates interpretability barriers that may impede clinician acceptance and patient trust. While several studies incorporated explainable AI methods such as Gradient-weighted Class Activation Mapping (Grad-CAM), none systematically evaluated their impact on clinical decision-making or patient outcomes (22). Complementary frameworks such as SHAP can also clarify feature contributions (e.g., tumor texture, volume, or metabolic intensity), and embedding these explanations into reporting/PACS could facilitate human AI collaboration in practice (23).

The demographic homogeneity observed across the included studies raises essential questions about AI generalizability and health equity (24). The underrepresentation of diverse patient populations in training datasets may perpetuate healthcare disparities, as AI models may perform suboptimally in underrepresented groups which is constant in other studies as well. This concern is particularly relevant in lung cancer, where significant racial and socioeconomic disparities in outcomes already exist. Ensuring equitable AI performance across diverse populations will require deliberate efforts to include representative datasets and conduct subgroup analyses during model development and validation.

Economic considerations represent another critical dimension of AI implementation that remains largely unexplored in the current literature (25). While the direct costs of AI systems are substantial, encompassing software licensing, hardware infrastructure, and integration expenses, the potential for cost savings through improved treatment selection and reduced unnecessary interventions may justify these investments. The economic burden of lung cancer treatment, exceeding $21 billion annually in the United States, suggests that even modest improvements in treatment response prediction could yield significant healthcare savings through optimized resource allocation and reduced treatment failures. Formal cost-effectiveness analyses were not reported across the included studies and should be incorporated prospectively.

The heterogeneity observed across studies in AI architectures, training methodologies, and validation approaches reflects the nascent state of the field and the absence of standardized evaluation frameworks. This variability complicates direct comparisons between AI systems and may contribute to the moderate statistical heterogeneity observed in our meta-analysis (I² values in the mild range). We found no evidence of a threshold effect based on Spearman’s correlation, and leave-one-out sensitivity analyses did not materially change the pooled estimates, supporting the robustness of the findings. Funnel plot symmetry and Egger’s test were consistent with a low likelihood of publication bias (p = 0.21), although selective reporting cannot be entirely excluded. Potential overlap of patient cohorts from the same institutions could not be wholly resolved and may have modestly inflated pooled sample size estimates. Finally, the protocol for this review was not prospectively registered, which introduces some risk of reporting bias despite our PRISMA-guided methods.

The temporal dynamics of treatment response prediction present additional complexity that warrants consideration. Traditional imaging-based response assessment typically occurs at predetermined intervals, often weeks to months after treatment initiation. AI models capable of earlier response prediction could enable more timely treatment modifications, potentially improving outcomes while minimizing exposure to ineffective therapies. However, the optimal timing for AI-assisted response prediction and its integration with existing clinical protocols requires further investigation.

Future research should prioritize prospective, multicenter validation studies that address the limitations identified in this meta-analysis. Such studies should incorporate diverse patient populations, standardized imaging protocols, and comprehensive economic evaluations to provide robust evidence for clinical implementation. Additionally, the development of hybrid human-AI decision support systems that leverage the complementary strengths of both approaches may offer superior performance compared to either modality alone. The findings of this meta-analysis position AI as a promising adjunct to radiologist interpretation rather than a replacement technology (26). The modest but consistent performance improvements observed across multiple studies suggest that AI can enhance human expertise while preserving the critical role of clinical judgment in cancer care. This collaborative approach may optimize the benefits of both human experience and machine precision, ultimately improving patient outcomes through more accurate and timely treatment response prediction.

## Strengths and limitations

This meta-analysis provides one of the most comprehensive evaluations comparing artificial intelligence (AI) and radiologist performance in predicting lung cancer treatment response. Key strengths include a large pooled sample size of 6,615 patients across 11 independent studies, which confers strong statistical power. The methodology adhered to PRISMA 2020 guidelines, incorporating a multi-database search strategy (PubMed, Embase, Scopus, Web of Science, and Cochrane Library) with clearly defined inclusion criteria. Multiple diagnostic performance metrics (sensitivity, specificity, accuracy, and risk difference) were analyzed using standardized statistical models in RevMan, with transparent visualization through forest plots. Independent dual-reviewer screening and data extraction enhanced methodological reliability, with inter-rater agreement quantified (Cohen’s κ = 0.84). In addition, subgroup analyses by imaging modality (CT, PET, MRI) and clinical context (e.g., advanced disease, EGFR-targeted therapy) provided clinically relevant insights.

Several limitations should also be acknowledged. First, the predominance of retrospective designs (10 of 11 studies) increases susceptibility to selection bias and limits external validity. Second, potential population overlap between studies from the same institutions could not be fully resolved due to limited reporting, potentially inflating pooled sample sizes. Third, heterogeneity in imaging modalities, AI architectures, and “treatment response” definitions may affect comparability and introduce threshold effects. Fourth, incomplete demographic reporting across studies limits assessment of equity and generalizability to diverse patient populations. Fifth, none of the included AI systems had regulatory clearance, restricting immediate clinical applicability. Sixth, cost-effectiveness analyses were absent, despite economic feasibility being critical for adoption in resource-limited healthcare systems. Seventh, interpretability tools were rarely incorporated, with limited application of explainable AI approaches such as Grad-CAM or SHAP. Finally, while efforts were made to minimize reporting bias, publication bias remains possible due to underreporting negative or neutral findings.

## Conclusion

This meta-analysis demonstrates that AI models achieve modest but statistically significant superiority over radiologists in predicting lung cancer treatment response, with pooled gains in sensitivity and accuracy, particularly in CT and PET imaging and in subgroups with advanced disease or EGFR-targeted therapy. These findings highlight AI’s potential as a valuable adjunct to human expertise, capable of enhancing diagnostic precision while maintaining comparable specificity and safety.

However, the predominance of retrospective studies, incomplete demographic reporting, heterogeneity in AI architectures, and lack of regulatory clearance limit the immediate generalizability of these results. Furthermore, few studies have incorporated explainable AI methods or assessed cost-effectiveness, which are factors critical for clinical adoption.

Future research should prioritize large-scale, multicenter, prospective trials that evaluate AI in real-world workflows, incorporate transparent and interpretable algorithms, assess equity across diverse populations, and address economic feasibility. AI integration should be conceptualized as augmenting, not replacing, radiologist judgment, fostering a multidisciplinary, patient-centered approach to lung cancer care. Additionally, the MRI-only subgroup did not show a significant AI advantage, and publication bias appeared unlikely based on Egger’s test (p = 0.21).

## Data Availability

The original contributions presented in the study are included in the article/**Supplementary Material**. Further inquiries can be directed to the corresponding author.

## Supplementary material

Supplementary Table 1 Full database-specific search strategies (through March 31, 2025).
