# Supplementary material for: Artificial intelligence versus radiologists in predicting lung cancer treatment response: a systematic review and meta-analysis
Source: Front Oncol. 2025 Oct 8;15:1634694. doi: 10.3389/fonc.2025.1634694 (PMC12540067; doi:10.3389/fonc.2025.1634694)
Supplement: Supplementary Table 1 — Full database-specific search strategies (through March 31, 2025). [file Table1.pdf]

Supplementary Table S1. Full database-specific search strategies (through March 31, 2025)

Database	Platform	Controlled Vocabulary	Full Search String	Date Range	Filters / Notes	ID
**PubMed/MEDLINE**	NLM	MeSH: *Lung Neoplasms*; *Artificial Intelligence*; *Machine Learning*; *Deep Learning*; *Treatment Outcome*; *Image Interpretation, Computer-Assisted*	((“Lung Neoplasms”[Mesh] OR “lung cancer” OR “non-small cell lung cancer” OR NSCLC OR “small cell lung cancer” OR SCLC) AND (“Artificial Intelligence”[Mesh] OR “Machine Learning”[Mesh] OR “Deep Learning”[Mesh] OR radiomics OR “Image Interpretation, Computer-Assisted”[Mesh] OR “computer-assisted” OR “convolutional neural network” OR CNN) AND (“Treatment Outcome”[Mesh] OR “treatment response” OR “response assessment” OR RECIST OR “pathologic response”) AND (radiologist* OR radiology) AND (predict* OR prognos* OR assess*))	Inception – 2025-03-31	Humans; exclude editorials, letters, non-comparative case reports; exclude conference abstracts without complete data	S1a

**Embase**	Ovid	Emtree: *lung cancer*; *non-small cell lung cancer*; *artificial intelligence*; *machine learning*; *deep learning*; *treatment outcome*	(exp lung cancer/ OR exp non small cell lung cancer/ OR (NSCLC OR SCLC OR lung neoplasm*).ti,ab.) AND (exp artificial intelligence/ OR exp machine learning/ OR exp deep learning/ OR radiomics.ti,ab. OR ("convolutional neural network*" OR CNN).ti,ab.) AND ("treatment response".ti,ab. OR exp treatment outcome/ OR RECIST.ti,ab. OR "response assessment".ti,ab. OR "pathologic response".ti,ab.) AND (radiologist*.ti,ab. OR radiology.ti,ab.) AND (predict*.ti,ab. OR prognos*.ti,ab. OR assess*.ti,ab.)	Inception – 2025-03-31	Exclude publication type "conference abstract"; prioritize human studies	S1b

**Scopus**	Elsevier	—	TITLE-ABS-KEY((("lung cancer" OR NSCLC OR SCLC OR "lung neoplasm*") AND ("artificial intelligence" OR "machine learning" OR "deep learning" OR radiomics OR "convolutional neural network" OR CNN) AND ("treatment response" OR "response assessment" OR RECIST OR "treatment outcome" OR "pathologic response") AND (radiologist* OR radiology) AND (predict* OR prognos* OR assess*)))	Inception – 2025-03-31	Excluded notes/letters (non-comparative); excluded incomplete conference papers	S1c

**Web of Science Core Collection**	Clarivate	—	TS=(("lung cancer" OR NSCLC OR SCLC OR "lung neoplasm*") AND ("artificial intelligence" OR "machine learning" OR "deep learning" OR radiomics OR "convolutional neural network" OR CNN) AND ("treatment response" OR "response assessment" OR RECIST OR "treatment outcome" OR "pathologic response") AND (radiologist* OR radiology) AND (predict* OR prognos* OR assess*))	Inception – 2025-03-31	Articles and reviews prioritized; “Meeting Abstracts” excluded unless full data available	S1d

**Cochrane Library**	Wiley	—	#1 MeSH descriptor: [Lung Neoplasms] explode all trees#2 ("lung cancer" OR NSCLC OR SCLC):ti,ab,kw#3 #1 OR #2#4 MeSH descriptor: [Artificial Intelligence] explode all trees#5 MeSH descriptor: [Machine Learning] explode all trees#6 ("artificial intelligence" OR "machine learning" OR "deep learning" OR radiomics OR "convolutional neural network" OR CNN):ti,ab,kw#7 #4 OR #5 OR #6#8 MeSH descriptor: [Treatment Outcome] explode all trees#9 ("treatment response" OR "response assessment" OR RECIST OR "treatment outcome" OR "pathologic response"):ti,ab,kw#10 #8 OR #9#11 (radiologist* OR radiology):ti,ab,kw#12 (predict* OR prognos* OR assess*):ti,ab,kw#13	Inception – 2025-03-31	Searched Reviews + CENTRAL (Trials); protocols excluded unless preliminary results reported	S1e

			#3 AND #7 AND #10 AND #11 AND #12			
**CINAHL**	EBSCOhost	CINAHL Headings: *Lung Neoplasms*; *Artificial Intelligence*; *Machine Learning*; *Treatment Outcomes*	((MH "Lung Neoplasms+" OR TI ("lung cancer" OR NSCLC OR SCLC) OR AB ("lung cancer" OR NSCLC OR SCLC)) AND (MH "Artificial Intelligence+" OR MH "Machine Learning+" OR TI (radiomics OR "deep learning" OR CNN) OR AB (radiomics OR "deep learning" OR CNN)) AND (MH "Treatment Outcomes+" OR TI ("treatment response" OR RECIST) OR AB ("treatment response" OR RECIST)) AND (TI (radiologist* OR radiology) OR AB (radiologist* OR radiology)) AND (TI (predict* OR prognos* OR assess*) OR AB (predict* OR prognos* OR assess*)))	Inception – 2025-03-31	Academic journals only; exclude dissertations and non-peer-reviewed sources	S1f

**Google Scholar**	Google	—	("lung cancer" OR NSCLC OR SCLC) AND ("artificial intelligence" OR "machine learning" OR radiomics) AND ("treatment response" OR RECIST) AND (radiologist OR radiology) AND (predict OR prognosis OR assess)	2015 – 2025-03-31	Grey literature and citation chasing; first ~200 results reviewed for relevance	S1g

Abbreviations: CNN, convolutional neural network; DL, deep learning; ML, machine learning; NSCLC, non-small cell lung cancer; RECIST, Response Evaluation Criteria in Solid Tumors; SCLC, small cell lung cancer.
